# Nitric Oxide Donor Modulates a Multispecies Oral Bacterial Community—An In Vitro Study

**DOI:** 10.3390/microorganisms7090353

**Published:** 2019-09-14

**Authors:** Takayuki Nambu, Dan Wang, Chiho Mashimo, Hugo Maruyama, Kosuke Kashiwagi, Kazushi Yoshikawa, Kazuyo Yamamoto, Toshinori Okinaga

**Affiliations:** 1 Department of Bacteriology, Osaka Dental University, 8-1, Kuzuha-Hanazono, Hirakata, Osaka 573-1121, Japan; mashimo@cc.osaka-dent.ac.jp (C.M.); maruyama@cc.osaka-dent.ac.jp (H.M.); 2 Department of Operative Dentistry, Graduate School of Dentistry, Osaka Dental University, 8-1, Kuzuha-Hanazono, Hirakata, Osaka 573-1121, Japan; wang-d@cc.osaka-dent.ac.jp; 3 Department of Fixed Prosthodontics, Osaka Dental University, 8-1, Kuzuha-Hanazono, Hirakata, Osaka 573-1121, Japan; kashiwagi-d.c@mopera.net; 4 Department of Operative Dentistry, Osaka Dental University, 8-1, Kuzuha-Hanazono, Hirakata, Osaka 573-1121, Japan; kazushi@cc.osaka-dent.ac.jp (K.Y.); yamamoto@cc.osaka-dent.ac.jp (K.Y.)

**Keywords:** oral microbiota, nitric oxide, sodium nitroprusside, 16S rRNA gene, high-throughput sequencing

## Abstract

The deterioration of human oral microbiota is known to not only cause oral diseases but also to affect systemic health. Various environmental factors are thought to influence the disruption and restoration of the oral ecosystem. In this study, we focused on the effect of nitric oxide (NO) produced by denitrification and NO synthase enzymes on dental plaque microbiota. Interdental plaques collected from 10 subjects were exposed to NO donor sodium nitroprusside (SNP) and then cultured in a specialized growth medium. Depending on the concentration of exposed SNP, a decrease in α-diversity and a continuous change in β-diversity in the dental plaque community were shown by sequencing bacterial 16S rRNA genes. We also identified eight operational taxonomic units that were significantly altered by NO exposure. Among them, the exposure of NO donors to *Fusobacterium nucleatum* cells showed a decrease in survival rate consistent with the results of microbiota analysis. Meanwhile, in addition to NO tolerance, an increase in the tetrazolium salt-reducing activity of *Campylobacter concisus* cells was confirmed by exposure to SNP. This study provides an overview of how oral plaque microbiota shifts with exposure to NO and may contribute to the development of a method for adjusting the balance of the oral microbiome.

## 1. Introduction

The human oral microbiota consists of more than 700 species of bacteria, with more than 100 species present in each individual [1,2,3]. Although its composition differs among individuals depending on the host’s environment, genetics, age, and lifestyle, the oral habitat is considered to house the most stable microbiota with the highest alpha diversity compared to that of other parts of the body [4,5]. The predominant members of the oral microbiota prevent the overgrowth of pathogenic microorganisms in the oral cavity not only by stimulating the immune system but also by forming the symbiotic biofilms [6]. The development of oral diseases is thought to be a consequence of the balance of the normally stable oral microbiota becoming unstable [5,7,8]. For example, frequent intake of carbohydrates leads to an ecosystem that contains many bacteria compatible with growth at low pH, thereby increasing the risk of caries [9,10,11]. Furthermore, excessive plaque accumulation is known to increase the risk of periodontal disease through changes to the microbiota that are dominated by anaerobic species that can cause inflammation [7,11,12]. In elderly people, it has been reported that differences in tongue microbiota may affect the onset of aspiration pneumonia [13,14]. However, due to the complexity of the microbial community and various environmental factors affecting the microbiota, it is very difficult to identify the individual species responsible for the observed physiological and pathological changes. For this reason, the mechanisms that disrupt the microbiota caused by various stressors and the nature of the oral ecosystem that leads to recovery from the disorder remain poorly understood.

Nitric oxide (NO) is one of the most important signaling molecules in human physiology and pathology, and is involved in multiple biological actions, such as vasodilation, angiogenesis, and neurotransmission [15,16,17]. In addition to NO production within the body, recent reports indicate that there are two parallel pathways for NO formation in the oral cavity [18,19]. The first is the nitric oxide synthase (NOS) pathway in which a family of NOS (neuronal NOS [nNOS], inducible NOS [iNOS], and endothelial NOS [eNOS]) catalyzes the oxidation of the amino acid L-arginine in the presence of molecular oxygen and several cofactors [20,21]. NO produced by iNOS in innate immune cells, including macrophages and neutrophils, acts as an antimicrobial agent to foreign pathogens [22]. While NO in physiological fluid has a short half-life of several seconds, NO possesses strong and broad-spectrum activity as an antibacterial agent, and forms equally reactive byproducts that cause both oxidative and nitrosative damage to microbial DNA, proteins, and exterior membrane structures [23]. The second pathway is microbial denitrification in the oral cavity. Dietary inorganic nitrate as a substrate is abundantly contained in green leafy vegetables and beets [24,25]. Ingested nitrate is efficiently absorbed into the circulation from the intestinal tract. Circulating nitrate is actively taken up by the salivary glands and concentrated in the saliva [26]. After the reduction of nitrate to nitrite by specific oral commensal bacteria, including *Rothia mucilaginosa*, *Neisseria flavescens*, and *Veillonella* spp. [8,19], the remaining nitrate and produced nitrite re-enter the enterosalivary loop. Since this nitrite can be further reduced to the potent vasodilator NO, dietary nitrate intake and the abundance of nitrate-reducing bacteria in the oral cavity represent routes to lower blood pressure and maintain and improve cardiovascular health [19]. These blood pressure-lowering effects of dietary nitrate are completely abolished with an anti-bacterial mouthwash [27,28]. In addition to cardiovascular function, a variety of nitrogen species including NO are formed and have potent anti-bacterial effects, when swallowed nitrite-containing saliva reaches the acidic gastric environment. In the oral cavity, part of the nitrite produced in the oral cavity is then further reduced to NO by the nitrite reductase activity of oral commensal bacteria or when exposed to the acidic conditions of the oral cavity. It has been reported that approximately 0.1 μM NO was detected in collected dental plaque [29]. NO had antimicrobial activity against some oral bacteria such as *Porphyromonas gingivalis*, *Aggregatibacter actinomycetemcomitans*, and *Actinomyces israelii* [30,31,32]. In addition, there seem to be some differences in NO sensitivity among oral bacterial species [30,31]. In terms of cytotoxicity, NO was substantially less toxic to human gingival fibroblasts than clinically relevant concentrations of chlorhexidine [30]. These findings suggest the potential usefulness of NO as a new platform for the development of periodontal disease prevention or treatment.

To date, although the bactericidal effect of NO on several oral bacteria has been investigated [30,31,32], how the bacterial microbiota structure shifts due to NO generated internally in the oral cavity or NO added from the outside has not yet been clarified. The purpose of this study was to investigate the effect of NO on the overall structure of a multispecies oral bacterial community and to obtain empirical data for controlling the community structure using nitrate and exogenous NO.

## 2. Materials and Methods

### 2.1. Dental Plaque Collection

Dental plaque was obtained using dental floss (Okina, Osaka, Japan) from ten healthy volunteers, aged between 24 and 52, who participated in this study. The characteristics in each subject are presented in Table S1. Each subject’s plaque was removed from the dental floss by pipetting with 100 μL phosphate-buffered saline (PBS) and transferred into a sterile plastic tube. Samples were immediately placed on ice and homogenized by repetitive pipetting with a narrow tip in an anaerobic chamber with 80% N_2_, 10% H_2_, and 10% CO_2_. Culture experiments were performed within 1 h after dental plaque collection.

The study protocol was reviewed and approved by the Osaka Dental University Medical Ethics Committee (approval no.: 111011; approval date: 3 December 2018). All experiments and data collections were performed in accordance with relevant guidelines and regulations. Written informed consent was obtained from all participants before inclusion in the study. The inclusion criterion was age >20 years. The exclusion criteria were as follows: self-reported presence of periodontitis or dental caries, current daily smoking, and treatment with local or systemic antibiotics within the past 1 month prior to participation. All participants were instructed to avoid eating or drinking for 1 h before plaque sampling.

### 2.2. Treatment of NO Donor and Culturing Plaque-Derived Microbial Microbiota

First, 30 μL aliquots of plaque suspensions were harvested, and stored at −80 °C as an initial sample. Ten microliters of plaque samples were mixed with 10 μL of the NO donor sodium nitroprusside (SNP, Sigma-Aldrich, Tokyo, Japan) solution in PBS to a final concentration of 0−100 mM and incubated under anaerobic conditions at 37 °C for 1 h. In order to determine the SNP concentration and incubation time tested, we referred to some reports on the bactericidal effect of NO [33,34,35]. After centrifugation, the supernatant containing SNP was carefully removed, and the remaining pellet was suspended in 1 mL of a specialized growth medium, SHI medium, containing 5% sheep blood, which is able to sustain similar bacterial populations to original oral microbiota [36,37,38,39]. The SNP-treated plaque samples were cultured at 37 °C for 20 h with shaking under anaerobic conditions. A 200 μL aliquot of the culture was centrifuged and the supernatant was discarded. The pellet samples were stored at −80 °C prior to DNA extraction within 1 week.

### 2.3. Sample Preparation for NGS and Sequencing

The identification and comparison of microbial communities was evaluated by next-generation sequencing of the 16S ribosomal RNA (rRNA) genes. DNA was extracted from frozen bacterial pellets using enzymatic, chemical, and mechanical lysis with a QIAamp UCP Pathogen Mini Kit (Qiagen, Hilden, Germany) and a Pathogen Lysis Tube S (Qiagen, Hilden, Germany) as previously described [40]. The purified genomic DNA was quantified with a Qubit dsDNA BR Assay Kit (Thermo Fisher Scientific, Waltham, MA, USA). Bacterial 16S ribosomal DNA amplification and library construction were performed according to the 16S Metagenomic Sequencing Library Preparation guide supplied by Illumina (part no. 15044223_B). Briefly, the V3-V4 region of 16S rRNA genes was amplified (25 cycles) via polymerase chain reaction (PCR) using primers 341F and 806R and Premix Ex Taq polymerase (Takara Bio, Otsu, Japan). The thermal cycling conditions were initial denaturation at 98 °C for 10 s, followed by 25 cycles at 98 °C for 10 s, 55 °C for 30 s, and 72 °C for 1 min. DNA integrity was verified by 1% agarose gel electrophoresis. After PCR purification using AGENCOURT AMPure XP Beads (Beckman Coulter, Brea, CA, USA), sequencing adapters containing 8 bp indices were incorporated into the 3’ and 5’ ends of the purified amplicons by PCR for 8 cycles under the conditions described above. After repurification with AMPure XP beads and pooling in an equimolar amount, 5% equimolar of PhiX DNA (Illumina, San Diego, California, USA) was added. The 16S rRNA gene amplicons were paired-end sequenced at 2 × 250 bp using a MiSeq Reagent Kit v2 (Illumina) on the Illumina MiSeq platform (Illumina). Sequencing was performed at the Oral Microbiome Center in Takamatsu, Japan.

### 2.4. Sequence Data Processing

The raw read sequences were analyzed with CLC Workbench software (version 10.0.1; Filgen, Nagoya, Japan) equipped with the Microbial Genomics Module plugin (version 2.0; Filgen). The paired end reads were merged into one high-quality representative by settings of CLC Workbench (mismatch cost = 1, minimum score = 25, gap cost = 4, maximum unaligned end mismatches = 5). The parameter settings for the quality trimming were as follows: trim using quality scores, limit = 0.05; trim ambiguous nucleotides, maximum number of ambiguities = 2. After fixed length trimming of 440 bases, operational taxonomic unit (OTU) clustering and taxonomic assignment were carried out with the reference sequences from the Human Oral Microbiome Database (HOMD, Version 14.51) at a level of similarity of 97% of OTU. To avoid analysis of spurious OTUs, only those with more than ten reads in at least one sample were kept.

### 2.5. Statistical Analyses

Data were analyzed in the R programming environment. The R-based Rhea pipeline [41] was used for data analysis yielding rarefaction curves, group significance, and taxonomic binning. After normalization of all samples by Rhea, the intrasample variation was calculated to quantify alpha diversity. We used Simpson’s index and Shannon’s index to estimate the diversity within a sample. Simpson places more weight on dominant species while Shannon focuses on richness and evenness. Richness gives the value of present OTUs within one sample. Generalized UniFrac distances were calculated to evaluate beta diversity (diversity between samples) [42]. Visualization of the multidimensional distance matrix in a space of two dimensions was performed by non-metric multidimensional scaling (NMDS). A PERMANOVA test was performed to determine statistically significant differences. *p*-values < 0.05 were regarded as statistically significant. Any *p*-values less than 0.05 are shown.

### 2.6. Quantitative PCR Analysis

For quantitative PCR (qPCR) quantification, the universal bacterial 16S rRNA primers, 339F (5′-ACTCCTACGGGAGGCAGCAGT-3′) and 514R (5′-ATTACCGCGGCTGCTGGC-3′), were used to estimate the concentration of total bacterial DNA. The 16S rRNA genes of *Actinomyces oris* MG1 were inserted into the vector plasmid pMD20-T using a Mighty TA-cloning Kit (Takara Bio, Shiga, Japan) and used as the real-time PCR control. The reactions were performed on a MiniOpticon RT-PCR detection system (Bio-Rad Laboratories Inc., Hercules, CA, USA) with QuantiFast SYBR Green PCR Master Mix (Qiagen GmbH, Hilden, Germany). Data was analyzed using the software BioRad CFX Manager version 1.5.

### 2.7. Bacterial Strains and Growth Conditions

*Fusobacterium nucleatum* subspecies *polymorphum* JCM 12990 and *Campylobacter concisus* ATCC 33237 were grown in modified Gifu anaerobic medium (GAM; Nissui Pharmaceutical, Tokyo, Japan) and Brucella medium (Becton Dickinson, Sparks, MD), respectively. Bacteria were cultivated at 37 °C under anaerobic conditions (nitrogen, 80%; carbon dioxide, 10%; and hydrogen, 10%).

### 2.8. Bactericidal Assay

*F. nucleatum* cells were precultivated anaerobically in 6 mL modified GAM medium for 20 h at 37 °C. Cells were washed with PBS buffer, resuspended, and then diluted to an optical density of 0.1 at 600 nm with the same buffer. NO donor, SNP or S-nitrosoglutathione (GSNO; Dojindo, Kumamoto, Japan), was added to the tubes to final concentrations in the range of 0–100 mM. After 1 h of incubation at 37 °C, the bacteria suspensions were immediately diluted with PBS and plated on a modified GAM plate using a spiral plater (Eddy Jet2, IUL Instruments, Barcelona, Spain). In the experiments for *C. concisus*, the same test was performed using Brucella medium (Becton Dickinson, Tokyo, Japan) instead of modified GAM medium. The inoculated plates were incubated anaerobically for 3 to 4 days at 37 °C. Colony counts were conducted using an automated plate counter (aCOLyte3, Synoptics, Cambridge, England).

### 2.9. Determination of Cellular Tetrazolium Salt-Reducing Activity

The Microbial Viability Assay Kit-WST (Dojindo, Kumamoto, Japan) uses reduced nicotinamide adenine dinucleotide (NADH) to reduce 2-(2-methoxy-4-nitrophenyl)-5-(2,4-disulfophenyl)-2H-tetrazolium monosodium salt (WST-8) to the water-soluble colored WST-8 formazan. Therefore, this kit can indirectly quantify cellular NADH. SNP was added to a final concentration of 0–100 mM in *F. nucleatum* subsp. *polymorphum* or *C. concisus* suspension prepared with Brucella medium at OD_600_ of 0.1. After anaerobic incubation at 37 °C for 1 h, samples were centrifuged at 13,000 *g* for 5 min, and the supernatants were carefully removed. The pellet was resuspended in an equal volume of Brucella medium and then diluted 10-fold with the same medium. After 190 μL of the diluted bacterial suspension was transferred into a 96-well microplate, 10 μL of WST-8 and an electron mediator solution were added, and the mixture was anaerobically incubated at 37 °C for 1 h. Immediately after incubation, absorbance at 450 nm was measured with a microplate reader SpectraMax M5 (Molecular Devices, Sunnyvale, CA, USA) to determine relative cellular reducing activity.

### 2.10. Availability of Data

The raw sequence data generated in this study are available in the DNA Data Bank of Japan (http://www.ddbj.nig.ac.jp/) under accession number DRA008873.

## 3. Results

### 3.1. NO Donor Partially Inhibited the Growth of Plaque Microbiota

**Figure 1:**
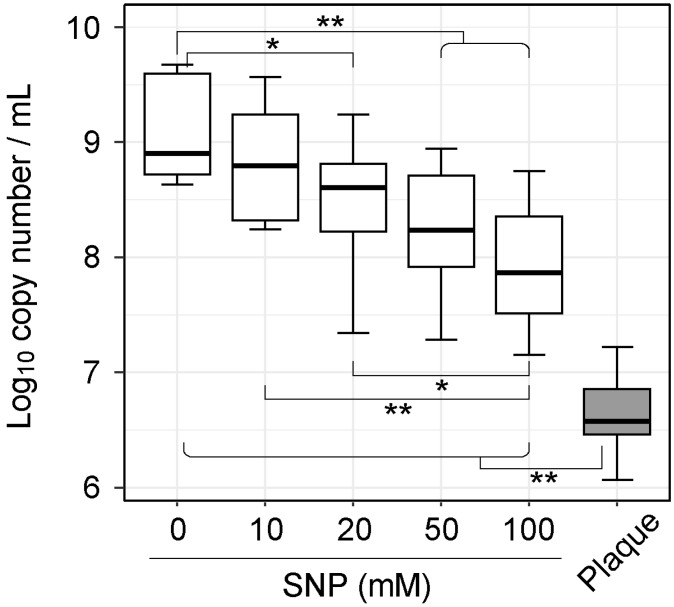
Total bacterial density in the initial plaque samples and in vitro cultures. For qPCR quantification, the universal bacterial 16S rRNA primers were used to estimate the concentration of total bacterial DNA. Horizontal lines indicate statistical significance according to the Wilcoxon rank-sum test (* *p* < 0.05, ** *p* < 0.01).

To investigate the effect of NO on dental plaque microbiota, plaque samples were obtained from ten healthy volunteers using dental floss. The samples were treated with NO donor, SNP, at final concentrations in the range of 0–100 mM for 1 h, and anaerobically cultured in SHI medium. As a preliminary experiment, we checked that approximately 30–240 μM of NO_2_, which is an index of NO production, was generated by adding 10–100 mM of SNP to the experimental environment (Figure S1). After 20 h of culture, DNA was extracted from the initial plaque and culture samples, and total bacterial density was first determined using 16S rDNA-gene quantification by qPCR. qPCR analysis revealed that the total bacterial density in greater than or equal to 20 mM SNP-treated samples was significantly lower compared to that in the non-SNP-treated sample (*p* < 0.05; Wilcoxon rank-sum test: Figure 1).

### 3.2. NO Donor Shifts Dental Plaque Microbiota in In Vitro Culture Condition

**Figure 2:**
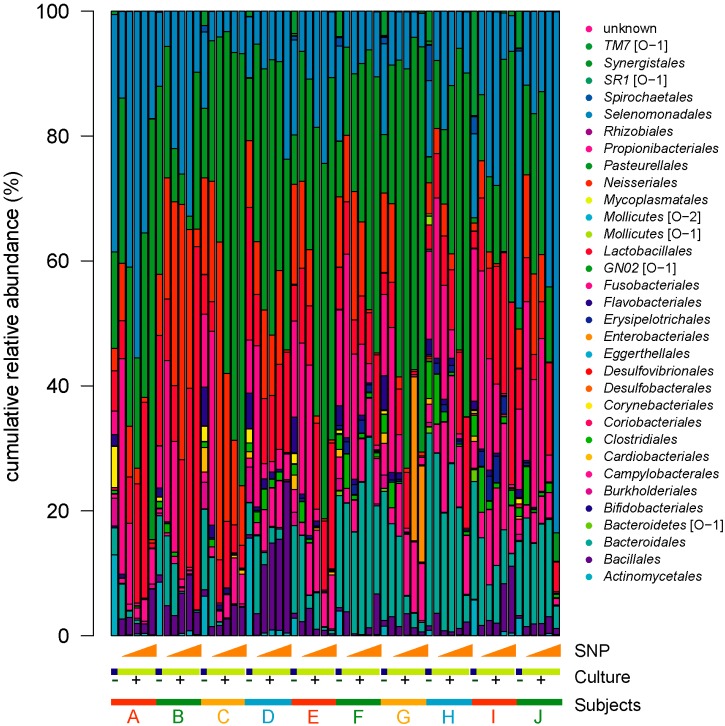
Bacterial community shift under sodium nitroprusside (SNP) exposure and in vitro culture. Stacked bar charts show taxonomic distribution at the order level. Each bar represents a single sample, with each color representing different bacterial taxa; sorted by individuals. The detailed information for each operational taxonomic unit (OTU) can be found in Table S2.

In order to examine shifts in plaque bacterial populations, the extracted DNA samples were subjected to high-throughput sequencing of the 16S rRNA gene. After paired-end assembly, quality filtering, chimera removal, and 97% OTU clustering, 4,156,072 sequences were clustered in 503 OTUs with an average number of 69,267 (±26,412 SD), a minimum of 33,180, and a maximum of 134,157 sequences per sample (Table S2). Rarefaction curves show that a plateau of species richness was achieved in approximately 30,000 reads per sample and indicate that a sufficient number of reads was obtained for 16S rRNA analyses (Figure S2). Stacked bar charts illustrating the relative proportion of order in the samples show that the plaque microbiota of each subject altered complexly by the addition of NO donor (Figure 2). However, we found no common changes in overall community structure between plaque samples due to NO exposure from this figure.

**Figure 3:**
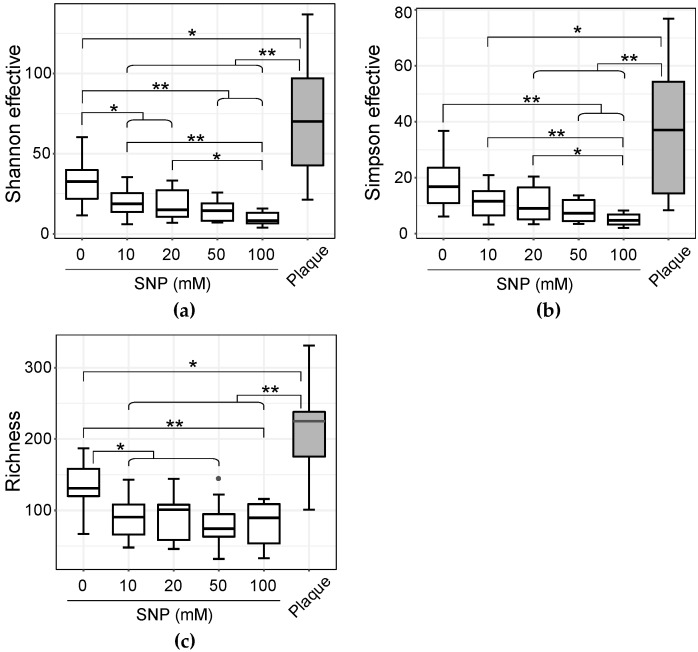
Alpha diversity indices in dental plaque and cultured samples. The R-based Rhea pipeline [41] was used for data analysis. The indices are plotted with three alpha diversity indicators: (**a**) Shannon effective; (**b**) Simpson effective; (**c**) species richness. *p*-values determined by paired Wilcoxon signed rank-sum test (* *p* < 0.05, ** *p* < 0.01).

**Figure 4:**
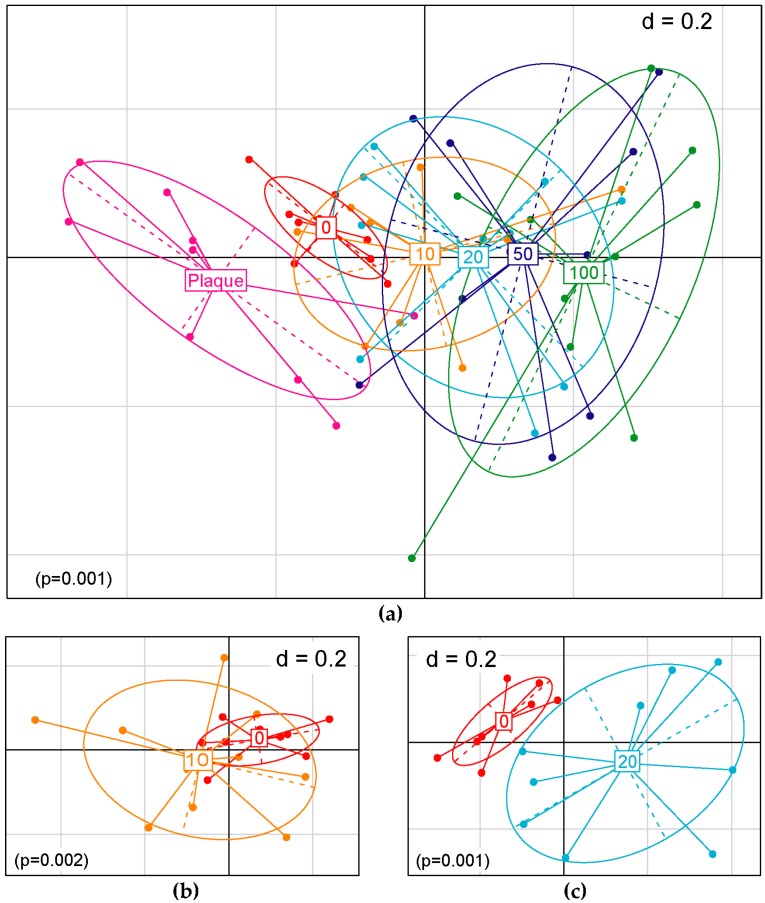
The effect of sodium nitroprusside (SNP) on beta diversity. Overall plaque microbiome composition was depicted by non-metric multidimensional scaling (NMDS) analysis. The plaque samples were treated with NO donor, SNP, at final concentrations in the range of 0–100 mM for 1 h. (**a**) All microbial communities were clustered according to treatment conditions. The relationships between untreated and 10 mM SNP-treated samples (**b**) and untreated and 20 mM SNP-treated samples (**c**) are extracted and depicted. The numbers in the squares indicate the final concentration (mM) of the added SNP. Samples on the first and second principal coordinates are plotted by nodes. Lines connect samples in the same groups, and colored circles cover the samples near the center of gravity for each group.

The intrasample diversity (alpha diversity) of the community significantly decreased with the addition of 10 mM or more SNP in both Shannon effective count and species richness (Figure 3a,c). In the Simpson effective count, a significant decrease was observed with the addition of 50 mM and 100 mM SNP (Figure 3b). In addition, it became clear that alpha diversity decreased depending on the concentration of added NO donor. When comparing the initial plaques with the samples cultured in SHI medium without SNP treatment, a significant difference was observed in species richness and the Shannon effective index, but no significant differences were observed in the Simpson effective index. The intercommunity diversity (beta diversity) also shifted in correlation with the concentration of SNP supplemented (Figure 4a). The 10 mM SNP-treated communities were significantly different but not clearly separated from their untreated counterparts in the principal coordinates analysis (PCoA) space, whereas the 20 mM SNP-treated communities were clearly separated from their untreated counterparts (Figure 4b,c).

### 3.3. NO Donor Alters the Relative Proportions of Some Specific Oral Bacteria

**Figure 5:**
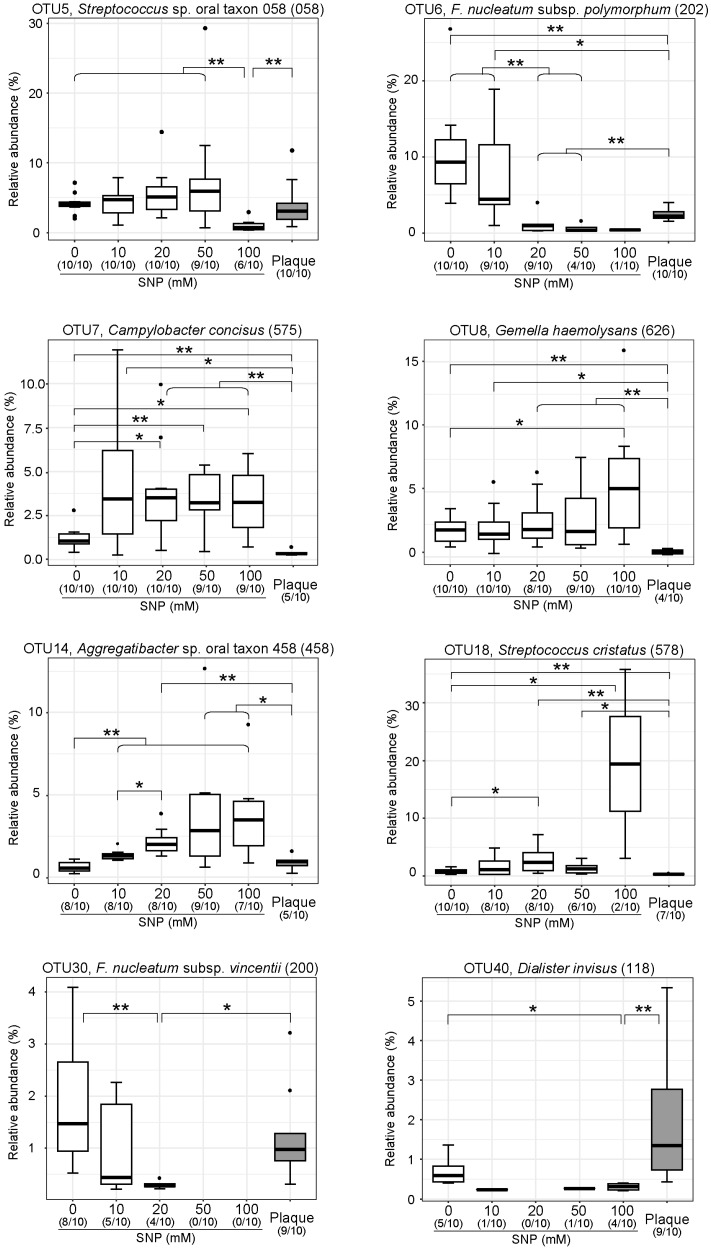
Relative abundance of the eight OTUs altered by SNP treatment. Differential abundance analysis was performed using the Wilcoxon rank-sum test at the OTU level (* *p* < 0.05, ** *p* < 0.01). Oral taxon IDs in HOMD are given in parentheses following bacterial names.

**Figure 6:**
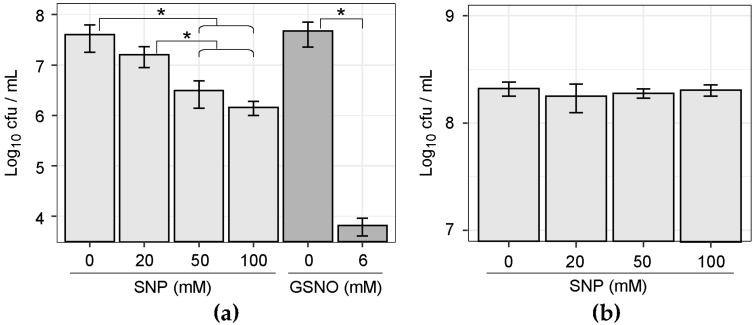
Effect of NO donor on the cell viability of bacterial strains. (**a**) Viability of *F. nucleatum* subsp. *polymorphum* growing in the presence or absence of NO donor SNP or S-nitrosoglutathione (GSNO). (**b**) Viability of *C. concisus* growing in the presence or absence of SNP. Values are expressed as the mean ± standard deviation, calculated from quadruplicate assays. *p*-values were calculated by Student’s *t*-test (* *p* < 0.05).

The results of this study indicate that the dental biofilm or plaque is a community composed of oral bacteria with different NO sensitivity. Although the analysis was performed using samples from only 10 subjects, eight OTUs with statistically significant differences were detected using the Wilcoxon rank-sum test (Figure 5). The relative abundance of *Streptococcus* sp. oral taxon 058, *F. nucleatum* subsp. *polymorphum*, *F. nucleatum* subsp. *vincentii*, and *Dialister invisus* were decreased, whereas the relative abundance of *Campylobacter concisus*, *Gemella haemolysans*, *Aggregatibacter* sp. oral taxon 458, and *Streptococcus cristatus* were increased depending on the concentration of NO donor supplemented. As for *F. nucleatum*, *F. nucleatum* subsp. *animalis* was also shown to decrease significantly by Fisher’s exact test (Figure S3). In fact, when the cultured *F. nucleatum* subsp. *polymorphum* was treated with SNP or another NO generator S-nitrosoglutathione (GSNO), a significant decrease in colony forming unit (CFU) count was confirmed (Figure 6a). In contrast, no decrease in CFU count could be observed when cultured *C. concisus* was treated with SNP (Figure 6b). Some bacterial species with increased relative abundance in the community, such as *C. concisus*, may have a mechanism that avoids the bactericidal effects of the produced NO.

### 3.4. Cellular WST-Reducing Activity was Induced by SNP Stimulation in C. concisus

**Figure 7:**
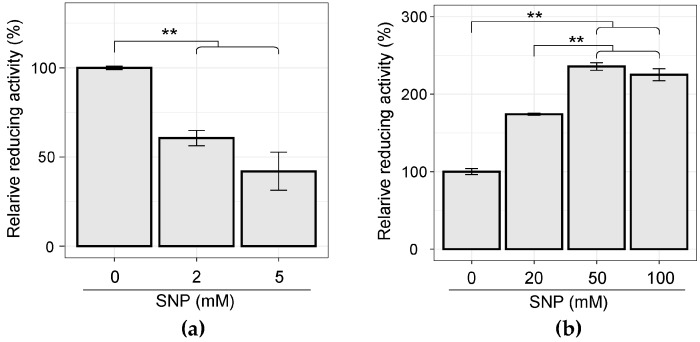
Relative WST-8-reducing activity of SNP-treated cells as determined by WST-8 reduction assay. Reduction of WST-8 to formazan by NADH in *F. nucleatum* subsp. *polymorphum* (**a**) and *C. concisus* (**b**) was measured at 450 nm using a microplate reader. Values are expressed as the mean and standard deviation, calculated from triplicate assays. *p*-values were calculated by Student’s *t*-test (** *p* < 0.01).

In order to clarify the cause of the NO resistance of *C. concisus*, the cellular WST-reducing activity was estimated by measuring the amount of WST-8 formazan produced from WST-8. When cultured *F. nucleatum* subsp. *polymorphum* was exposed to SNP, a sudden decrease in the relative reducing activity was observed as the SNP concentration increased (Figure 7a). In contrast, a significant increase in the absorbance value indicating the amount of WST-8 formazan could be confirmed when cultured *C. concisus* was treated with SNP (Figure 7b). This increased cellular WST-reducing activity may be responsible for SNP resistance in *C. concisus*.

## 4. Discussion

The complex symbiotic interaction between a balanced oral microbiome and the host aids in maintaining oral and systemic health. Harmful changes in oral microbiota balance (dysbiosis) can lead to dental caries, periodontal disease, and various systemic diseases, including cardiovascular disease, rheumatoid arthritis, colorectal cancer, pneumonia, and diabetes [5,6,8,43]. In order to elucidate the nature of oral ecosystems, several studies have been conducted to investigate the response of oral microbiota using environmental factors, such as nitrate, erythritol, and arginine [44,45,46]. In this study, we investigated the effect of NO, which is known to occur in the oral cavity and is one of the environmental factors that may shift the oral microbiota, on dental plaque using a simple system for in vitro culture. NO exposure to plaque samples caused a decrease in total bacterial density after SHI culture, suggesting that NO inhibited the growth of a considerable part of the plaque bacteria. This study also showed that dental plaque is composed of bacterial groups differing in NO sensitivity, and that the microbiota structure may change depending on NO concentration. Under the conditions of this experiment, the suspended plaque was exposed to SNP or NO donor for 1 h; however, it is assumed that plaque comes in contact with NO at relatively low concentrations for a long time or at high concentrations locally in the actual oral environment. Moreover, as mentioned above, since the bacterial composition of the plaque of each individual is different, the response patterns of ecosystems also appear to be different for each individual [8]. Because of the difference in environmental conditions in each situation, the results of this experiment do not completely reflect the oral ecosystem. However, we believe that this article has provided an overview of how the dental plaque microbiota shifts when exposed to NO.

We showed that oral commensal bacterium *C. concisus* is resistant to NO toxicity, and its abundance in the microbiota was increased by NO treatment to plaque and in vitro culture. Further, it was demonstrated that the amount of NADH, or the WST-reducing activity, of *C. concisus* cells was enhanced in response to NO treatment. In fact, *Campylobacter* has been reported to be able to colonize various environments from the oral cavity to the intestinal tract using a mechanism to cope with high nitrosative stress like that caused by *Helicobacter* [47]. *C. jejuni*, a major human enteric pathogen, passes through the stomach, where there is a high concentration of NO due to the acid-induced chemical degradation of salivary nitrite, and then enters the small intestine [48]. From the above discussion, it is speculated that, even in the dental plaque, some bacteria are killed by nitrosative stress, while some bacteria survive by expressing various detoxification enzymes and expand the ratio even under a reactive nitrogen species (RNS) stress environment. In *C. jejuni*, the presence of NADH is necessary for the function of the globin proteins responsible for NO elimination [49]; hence, NADH accumulation may have occurred in response to the addition of NO donors. The proportion of *Campylobacter* species has been reported to be relatively high in periodontal lesions [47]. This phenomenon may also be explained by the fact that *Campylobacter* avoids the NO produced during inflammation at the periodontal lesions, resulting in an increase in the ratio. In addition, it was reported that *C. concisus* have enzymes that reduce NO_2_ to NH_3_ or N_2_O [50,51]. Our result suggests that secondary fluctuations in oral microbiota may be induced by the production of NH_3_ and N_2_O produced from NO_2_, which is generated by the oxidation of NO. For further elucidation of the nature of the oral microbiome, an integrated analysis of the oral microbiota, including secondary shifts, in conjunction with microbiota and metabolome analyses may be necessary.

In this study, we found that *F. nucleatum*, regardless of the subspecies classification, was highly sensitive to NO treatment and significantly reduced its abundance in the microbiota following treatment in dental plaque and in vitro culture. This means that the ratio of *F. nucleatum* in dental plaque can be selectively reduced by NO exposure. *F. nucleatum* has an important role in oral biofilm maturation, serving as a coaggregation bridge organism that links early colonizing commensals and late pathogenic colonizers [52]. In addition to its association with periodontal disease, *F. nucleatum* has been suggested to be associated with the development and progression of colorectal cancer (CRC) [52,53,54,55,56]. In addition, exposure to low concentrations of NO has been reported to cause inhibition of the oral biofilm formation of *F. nucleatum* [57]. If it is possible to control the ratio of *F. nucleatum* in the oral cavity by controlling the production of NO, it may be possible to reduce the progression of periodontitis and colon cancer.

In this experiment, interdental plaque was used as the subject to be treated with the NO donor. It has been reported that interdental plaques contain relatively large amounts of *F. nucleatum* [58], and in fact, significant reductions of the three subspecies of *F. nucleatum* could be confirmed. However, although the bactericidal effect of NO against *Porphyromonas gingivalis* and *Aggregatibacter actinomycetemcomitans* has previously been reported [30,32], statistical analysis in this study failed to show vulnerability against this NO toxicity. The major cause is that the OTUs corresponding to these bacteria were hardly detectable in the samples, since interdental plaques from young subjects with healthy periodontal tissues were used. In fact, while *A. actinomycetemcomitans* was not detected in any of the samples, *P. gingivalis* was detected in 2 out of 10 subjects, and a decrease in the abundance ratio was observed by SNP treatment (data not shown). In the future, by increasing the number of samples used for analysis and analyzing samples from other regions, such as the subgingival plaques of periodontitis patients and tongue coatings, it is expected that the oral bacterial species that respond to NO can be identified exhaustively.

In addition to *C. concisus* and *F. nucleatum*, we identified six bacteria that changed significantly with NO exposure (Figure 5). Among them, *Gemella haemolysans* and *Aggregatibacter* sp. oral taxon 458 appear to be clearly resistant to NO. *G. haemolysans* is known as one of the bacterial species frequently co-isolated from the lungs of patients with cystic fibrosis (CF) [59]. In CF patients, the expression of low levels of molecules involved in biological defense such as nitric oxide or glutathione has been shown to decrease, so the ability of this bacterium to resist NO may be involved in the chronic infection of CF patients. The 16S rRNA sequence of *Aggregatibacter* sp. oral taxon 458 was closely related to that of *Aggregatibacter aphrophilus* as a result of BLAST analysis (data not shown). Although *A. aphrophilus* has been shown to be associated with infective endocarditis and brain abscess, the question remains as to whether there is a link between the NO resistance of this bacterium and the development of these infections [60].

Because microbial resistance to NO has not been observed based on its multimechanistic antimicrobial activity, recent studies have focused on the utility of exogenous NO for the treatment of infectious diseases as an antimicrobial and anti-biofilm agent [61,62,63]. In fact, while the efficacy of NO as an antimicrobial agent has been demonstrated against medically important bacterial organisms (e.g., *Escherichia coli*, *Pseudomonas aeruginosa*, and *Staphylococcus aureus*), bactericidal effects on periodontal pathogens have also been reported [30,31,32,64]. In terms of cytotoxicity, NO was substantially less toxic to human gingival fibroblasts than clinical concentrations of chlorhexidine [30]. Additionally, many NO donors and NO-releasing macromolecular scaffolds have been developed to facilitate the continuous delivery of exogenous NO [65]. While exogenous NO has been studied as an attractive antimicrobial agent that can kill bacteria directly, we demonstrated that NO can function as a molecule that modulates oral microbial ecology. Developing a technique to release appropriate concentrations of NO and/or to control the generated NO may contribute to the improvement of human health through the maintenance and restoration of a balanced oral microbiome.

## 5. Conclusions

In this study, NO-induced compositional shifts in oral microbiota were observed for the first time by combining in vitro culture techniques with a high-throughput sequence. In addition, we were able to identify several bacterial OTUs that respond to NO exposure in common with each subject’s ecosystem. In the future, it may be possible to develop a method to maintain a balanced oral microbiome through investigating the responsiveness to NO and its secondary metabolites in the ecosystem of other parts in the oral cavity.

## Supplementary Materials

The following are available online at https://www.mdpi.com/2076-2607/7/9/353/s1.
