# Supplementary material for: Nitric Oxide Donor Modulates a Multispecies Oral Bacterial Community—An In Vitro Study
Source: Microorganisms. 2019 Sep 14;7(9):353. doi: 10.3390/microorganisms7090353 (PMC6780529; doi:10.3390/microorganisms7090353)
Supplement: Supplementary file 1 [file microorganisms-07-00353-s001.zip › microorganisms-587004-supplementary figures.docx]

Supplementary


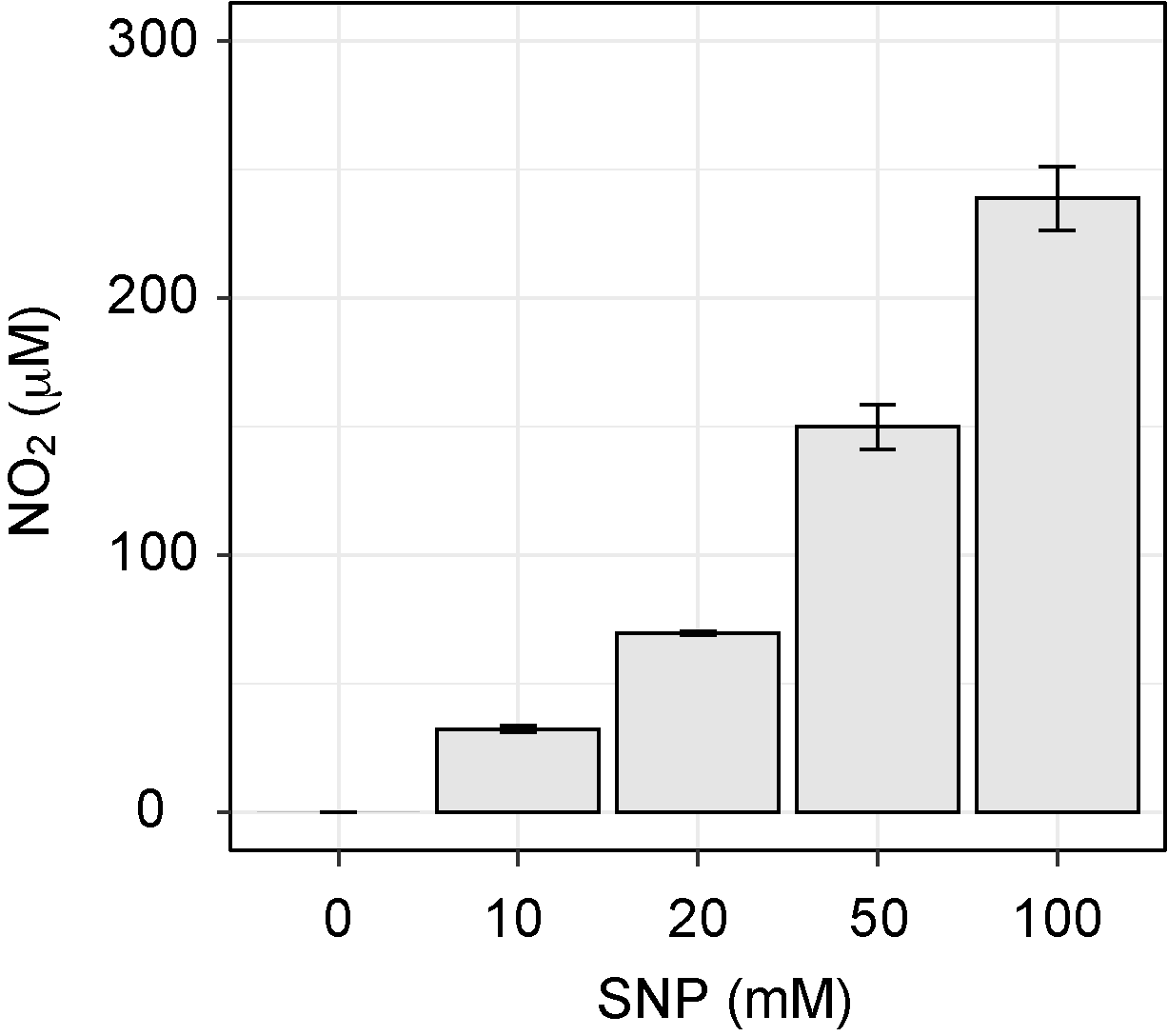


Figure S1: Nitrite production by SNP.

SNP was dissolved in PBS and incubated at 37 °C under anaerobic condition for 1 hour. The concentration of nitrite produced by SNP was determined using high-performance liquid chromatography analyzer (ENO-30, EICOM, Japan). The results were expressed as mean values (n=3) with their standard deviations.


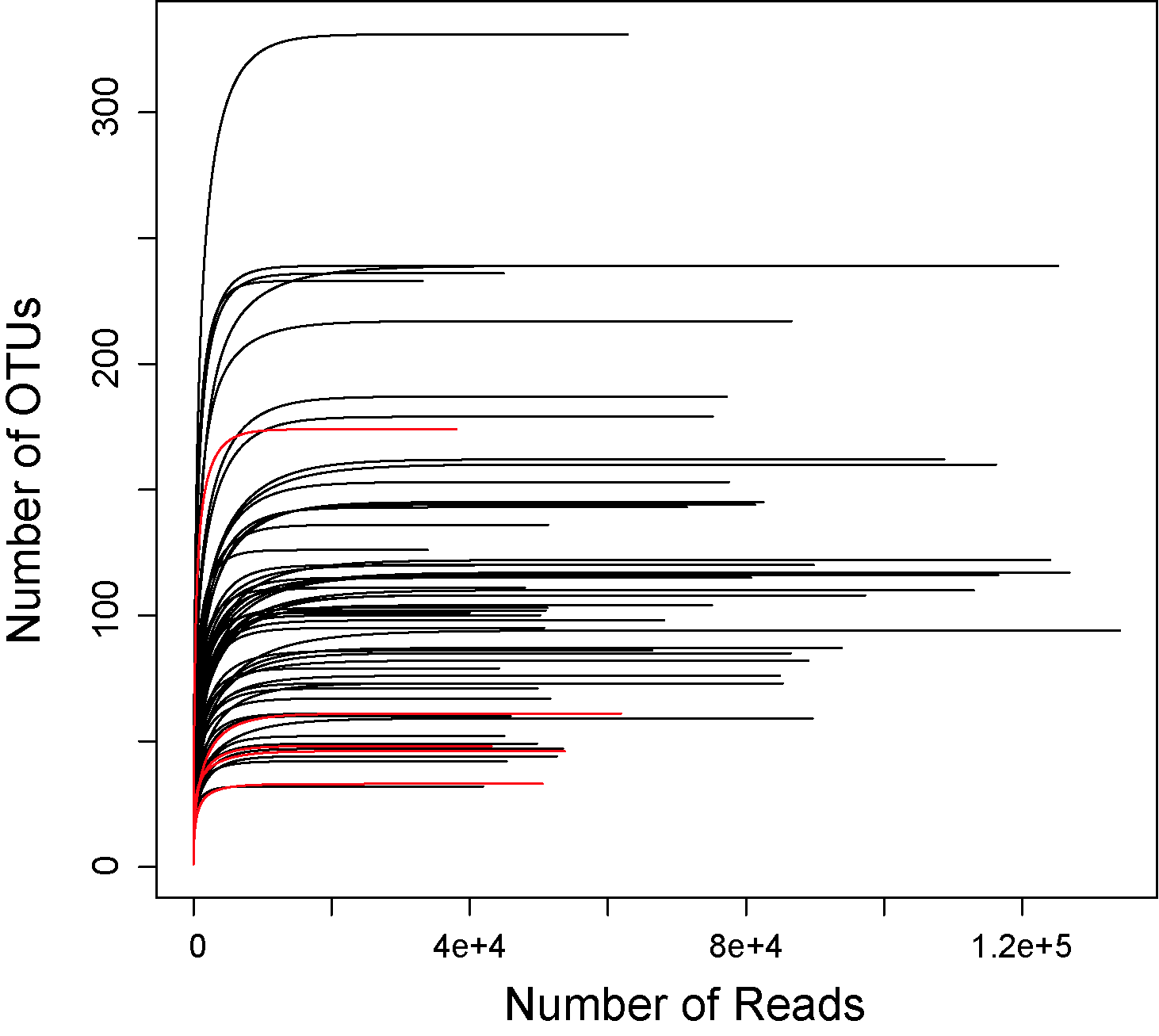


Figure S2: Rarefaction curve of all samples included in the study. The red lines indicate the samples with the steepest rarefaction curves.


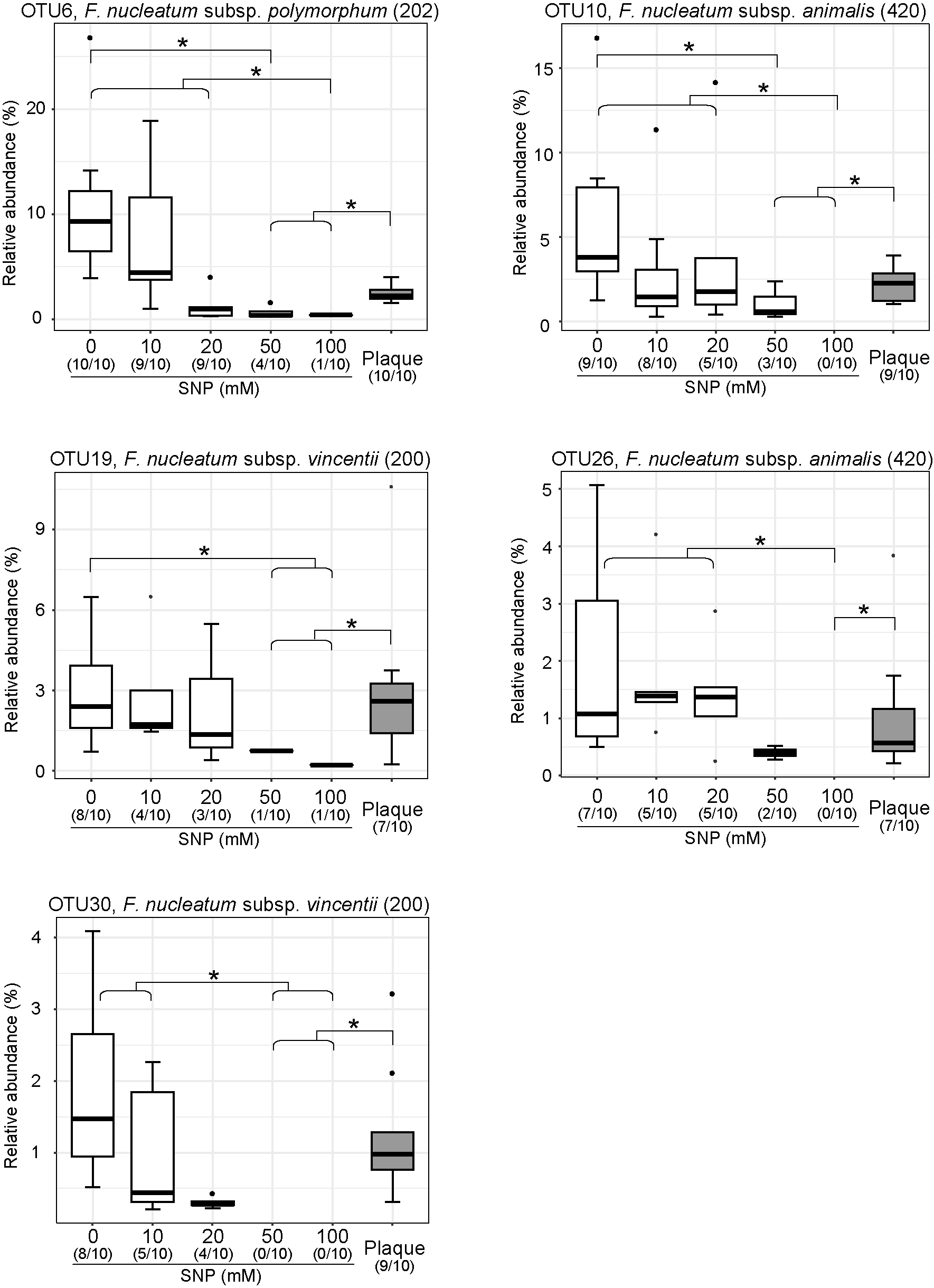


Figure S3: Shifts of relative abundances of *F. nucleatum* subspecies by SNP exposure.

The five OTUs of *F. nucleatum* that shifted significantly with the Fisher's Exact Test are shown (* < 0.05, ** < 0.01). As for *F. nucleatum* subsp. *polymorphum* (OTU6) and *F. nucleatum* subsp. *vincentii* (OTU30), statistically significant differences were also detected with Wilcoxon rank-sum test (see Fig. 5).
